# How Rainforest Conversion to Agricultural Systems in Sumatra (Indonesia) Affects Active Soil Bacterial Communities

**DOI:** 10.3389/fmicb.2018.02381

**Published:** 2018-10-10

**Authors:** Dirk Berkelmann, Dominik Schneider, Martin Engelhaupt, Melanie Heinemann, Stephan Christel, Marini Wijayanti, Anja Meryandini, Rolf Daniel

**Affiliations:** ^1^Genomic and Applied Microbiology and Göttingen Genomics Laboratory, Institute of Microbiology and Genetics, Georg-August-University, Göttingen, Germany; ^2^Department of Biology, Faculty of Mathematics and Natural Sciences IPB, Bogor Agricultural University, Bogor, Indonesia

**Keywords:** 16S rRNA gene transcripts, soil bacterial communities, rainforest conversion, active bacterial communities, oil palm plantation, Sumatra

## Abstract

Palm oil production in Indonesia increased constantly over the last decades, which led to massive deforestation, especially on Sumatra island. The ongoing conversion of rainforest to agricultural systems results in high biodiversity loss. Here, we present the first RNA-based study on the effects of rainforest transformation to rubber and oil palm plantations in Indonesia for the active soil bacterial communities. For this purpose, bacterial communities of three different converted systems (jungle rubber, rubber plantation, and oil palm plantation) were studied in two landscapes with rainforest as reference by RT-PCR amplicon-based analysis of 16S rRNA gene transcripts. Active soil bacterial communities were dominated by *Frankiales* (*Actinobacteria*), subgroup 2 of the *Acidobacteria* and *Alphaproteobacteria* (mainly *Rhizobiales* and *Rhodospirillales*). Community composition differed significantly between the converted land use systems and rainforest reference sites. *Alphaproteobacteria* decreased significantly in oil palm samples compared to rainforest samples. In contrast, relative abundances of taxa within the *Acidobacteria* increased. Most important abiotic drivers for shaping soil bacterial communities were pH, calcium concentration, base saturation and C:N ratio. Indicator species analysis showed distinct association patterns for the analyzed land use systems. Nitrogen-fixing taxa including members of *Rhizobiales* and *Rhodospirillales* were associated with rainforest soils while nitrifiers and heat-resistant taxa including members of *Actinobacteria* were associated with oil palm soils. Predicted metabolic profiles revealed that the relative abundances of genes associated with fixation of nitrogen significantly decreased in plantation soils. Furthermore, predicted gene abundances regarding motility, competition or gene transfer ability indicated rainforest conversion-induced changes as well.

## Introduction

Palm oil and rubber production play a crucial role for the economy in several countries. Especially in Indonesia, as one of the top producers of palm oil and rubber, conversion of natural systems to agricultural systems almost doubled from 2000 to 2009 (Angelsen, 1995; McCarthy, 2010; Oosterveer, 2015; Ivancic et al., 2016). In most cases, primary and secondary rainforests were converted to managed cash crop systems. Since the major part of the global biodiversity is inherited by tropical forests, the enormous biodiversity harbored by Indonesians rainforests was reduced drastically during this the process. Consequently, deforestation and conversion to agricultural systems in tropical regions is considered the biggest threat to global biodiversity. This affects not only animal and plant communities, but also microbial communities and tropical ecosystem functions as well (Donald, 2004; Sodhi et al., 2004; Koh et al., 2011; Wilcove et al., 2013; Barnes et al., 2014).

Microbial and, in particular, bacterial communities drive almost all biogeochemical cycles and are involved in nutrient cycling in soils (Fierer and Jackson, 2006; Delmont et al., 2011, 2012). Therefore, soil bacteria are closely connected to the lifestyles of other organisms and nutrient availability itself. Additionally, it was suggested that the community response of soil bacteria toward changes in nutrient availability and plant diversity follows predictable patterns (Waldrop et al., 2000; Leff et al., 2015). In soil, the involvement of microbes in nutrient cycling is crucial for soil fertility and therefore for plant growth and growing cash crops (Bhardwaj et al., 2014; Lynch, 2015). In the last years, several studies investigated the effects of logging and land transformation on soil bacterial communities and confirmed that rainforest conversion to oil palm or rubber plantations has severe impacts on soil prokaryotic diversity and composition (Lee-Cruz et al., 2013; Schneider et al., 2015; Kerfahi et al., 2016). Soil bacterial and archaeal diversity increased with increasing land use intensity and biomass decreased with pH and C:N ratio, which were identified as main abiotic drivers of bacterial community formation (Allen et al., 2015; Schneider et al., 2015). It was also shown that some procedures of rainforest exploitation, like logging, appear reversible, which makes further research on the topic even more crucial (Tripathi et al., 2012; Lee-Cruz et al., 2013; Kerfahi et al., 2016).

The results of most previous studies were obtained by DNA-based 16S rRNA gene analyses representing the entire community whereas effects on activity and functional distribution of prokaryotic groups have rarely been addressed. Since microorganisms can be abundant while remaining inactive or dormant and even can have different numbers of ribosomal operons, the actual impact and importance for the corresponding communities or ecosystem can be biased (Urich et al., 2008; Větrovský and Baldrian, 2013; Wemheuer et al., 2014). To avoid this issue, analyses based on RNA are required as well. Studies that aim to analyze bacterial activity by using 16S rRNA transcripts are well established for marine environments (Wemheuer et al., 2012, 2014; Rodríguez-Blanco et al., 2013; Zhang et al., 2014; Stibal et al., 2015). In soil and other terrestrial environments, however, only a limited number of RNA-based studies are available, which address the effects on land use conversion and showed that entire community analysis alone can lead to false conclusions regarding community activity (Foesel et al., 2014; Herzog et al., 2015; Mueller et al., 2016; Ragot et al., 2016).

As part of the “Ecological and Socioeconomic Functions of Tropical Lowland Rainforest Transformation Systems (Sumatra, Indonesia)” (EFForTS) collaborative research center, we investigated the impact of rainforest conversion on the active soil bacterial community and deduced their functional responses. This study is one of the first that investigates the effect of rainforest transformation to rubber and oil palm plantations on active bacterial soil communities in Indonesia.

Three different agricultural systems comprising intensively managed oil palm plantations, rubber plantations and jungle rubber were studied and compared to secondary rainforest in two different landscapes in Southwest Sumatra, Indonesia. Based on large-scale amplicon-based analysis of 16S rRNA transcripts, changes in the active bacterial communities were analyzed and correlated with biotic and abiotic factors. Besides investigating the active bacterial community composition and diversity, we also investigated effects on functional traits and compared our results to previous DNA-based studies.

As a guideline we followed three hypotheses: (a) Rainforest transformation leads to no significant changes of soil bacterial diversity, whereas (b) active bacterial community structure and composition is significantly affected. Furthermore, we hypothesized that (c) for rainforest and transformed land use systems specific indicator species can be detected and predicted metabolic profiles show significant functional shifts of the active bacterial community.

## Materials and Methods

### Sampling and Sample Treatment

**FIGURE 1:**
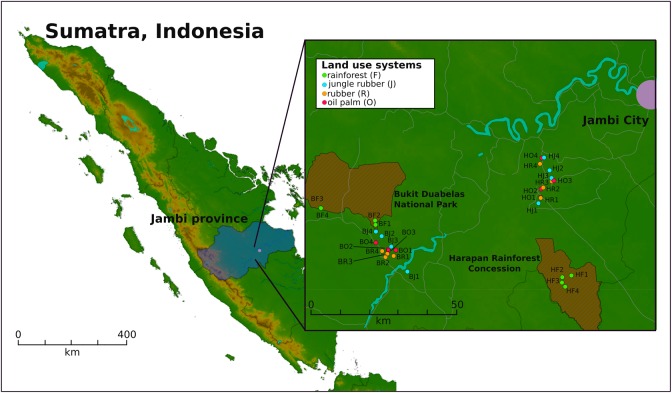
Sampling sites on Sumatra, Indonesia. Two landscapes within the province of Jambi were studied. Four core plots per land use system were analyzed, with three subplots per core plot in each landscape. The landscapes Bukit Duabelas and Harapan are indicated by “B” and “H” in the Plot ID. The conservation areas “Bukit Duabelas National Park” and “Harapan Rainforest Concession” are displayed as cross-hatched brown areas.

Two landscapes in southwest Sumatra (Indonesia), the Harapan Rainforest Concession (H) and Bukit Duabelas (B), were selected for sampling (**Figure 1**). Sampling was conducted on the plots as described by Schneider et al. (2015). Soil texture differed with primarily loam Acrisol soils in Harapan and clay Acrisol soils in Bukit Duabelas. Both landscapes harbored secondary rainforest (named “BF” and “HF”) and three different land use systems representing different land use intensities, which derived from rainforest conversion. The agricultural systems comprise oil palm plantations (monocultures of *Elaeis guineensis*; designated “BO” and “HO”), rubber plantations (monocultures of *Hevea brasiliensis*; designated “BR” and “HR”) and rubber agroforest (designated “BJ” an “HJ”). The latter represents a traditional agroforestry system known as “jungle rubber” in which rubber trees are planted in secondary rainforest without fertilizer or liming input. Each land use system consisted of four core plots in each landscape, with three subplots (five by five meters) per core plot, resulting in 96 subplot samples out of the 32 core plots in total. A soil corer was used to take three cores at each subplot of the upper seven cm of top soil and a diameter of five cm. The three soil samples per subplot were homogenized and coarse roots and stones (>5 mm) were removed. To prevent RNAs from degradation, RNAprotect Bacteria Reagent was applied to the samples as recommended by the manufacturer (Qiagen, Hilden, Germany). Samples were stored at -80°C until further use.

Age of rubber trees ranged from 15 to 40 and 6 to 16 years in jungle rubber and rubber plantations, respectively. In oil palm plantations, the age of the tress varied between 8 to 15 years (Kotowska et al., 2015). Management for the two plantation types included application of herbicides every 6 months and addition of inorganic NPK fertilizer [100–300 kg ha^-1^ yr^-1^ in rubber plantations and 300–600 kg ha^-1^ yr^-1^ in oil palm plantations (Kotowska et al., 2015)]. All soil parameters [pH, P, N, C, C:N ratio, Al, Ca, Fe, K, Mg, Mn, Na, effective cation exchange capacity (ECEC) and base saturation] were retrieved from Allen et al. (2015).

### RNA Isolation, cDNA Synthesis, and 16S rRNA Amplification

To analyze the active part of the bacterial communities, RNA was isolated from all 96 samples by using the MoBio PowerSoil RNA Isolation Kit (MO BIO Laboratories, Hilden, Germany). Isolation was initiated by using 0.5 g sample material. Residual DNA was removed by treatment with Turbo DNase as suggested by the manufacturer (Applied Biosystems, Darmstadt, Germany). The reaction mixture was subsequently purified and concentrated by using the RNeasy MinElute Cleanup Kit as recommended by the manufacturer (Qiagen). To verify the complete removal of DNA, a PCR reaction targeting the 16S rRNA gene was performed as described by Schneider et al. (2015). RNA yields were estimated by employing a Qubit^®^ Fluorometer as recommended by the manufacturer (Thermo Fisher Scientific, Waltham, MA, United States).

RNA was converted to cDNA by using the SuperScript^®^ III Reverse Transcriptase (Thermo Fisher Scientific, Waltham, MA, United States). As described by Wemheuer et al. (2015), a specific primer for the conserved region downstream to the targeted bacterial 16S rRNA gene region (5^′^-CCGTCAATTCMTTTGAGT-^′^) was used for cDNA synthesis. The reaction mixture (20 μl final volume) contained 4 μl of fivefold reaction buffer, 500 μM of each deoxynucleoside triphosphate, 5 mM DTT, 1 μM reverse primer, 1 U RiboLock^TM^ RNase Inhibitor (Thermo Fisher Scientific, Schwerte, Germany) and 200 U of reverse transcriptase. The solution was incubated at 55°C for 1 h and subsequently inactivated by incubation at 70°C for 15 min. To remove residual RNA in the RNA/DNA hybrids, 2.5 U RNase H (Thermo Fisher Scientific, Schwerte, Germany) was added and incubated at 37° for 15 min followed by an additional inactivation at 65°C for 10 min. Obtained cDNA was subsequently used for amplification of the hypervariable V3 to V5 regions of the 16S rRNA transcript [Forward primer: V3for_B 5^′^-CGTATCGCCTCCCTCGCGCCATCAG-MID-TACGGRAGGCAGCAG-3^′^ (Liu et al., 2007) reverse primer: V5rev_B 5^′^-CTATGCGCCTTGCCAGCCCGCTCAG-MID-CCGTCAATTCMTTTGAGT-3^′^ (Wang and Qian, 2009)]. The following thermal cycling scheme was used for amplification of partial bacterial 16S rRNA: initial denaturation at 98°C for 5 min, 25 cycles of denaturation at 98°C for 45 s, annealing for 45 s at 65°C, and extension at 72°C for 30 s, followed by a final extension period at 72°C for 5 min. All amplicon PCR reactions were performed in triplicate and pooled in equimolar amounts for sequencing. The Göttingen Genomics Laboratory determined the sequences of the 16S rRNA amplicons by using a 454 GS-FLX sequencer and Titanium chemistry following the instructions of the manufacturer (Roche, Mannheim, Germany) for amplicon sequencing.

### Analysis of 16S rRNA Transcripts

Raw sequences were processed and analyzed using QIIME 1.9.1 (Caporaso et al., 2010). Sequences with lengths below 300 and over, 1000 bp, quality scores below 25 and homopolymer stretches of more than 8 bp were removed by employing *split_libraries.py*. Denoising was performed with Acacia (default settings), chimera removal with UCHIME and reverse primer removal with cutadapt (Edgar et al., 2011; Bragg et al., 2012).

Determination of operational taxonomic units (OTUs) was performed at genetic divergence of 3% by employing the *pick_open_reference_otus.py* script with the SILVA NR SSU 128 database as reference. Taxonomic classification was performed with the same reference database and *parallel_assign_taxonomy_blast.py* script. OTU tables were created by employing *make_otu_table.py*. Further polishing including removal of singletons, chloroplast sequences, unclassified OTUs and extrinsic domain OTUs) was carried by employing *filter_otu_table.py*. Comparison of samples was performed by creating subsamples with identical sequence numbers (6,650 per subsample). Rarefaction estimates were done by *alpha_rarefaction.py*. Diversity indices, Shannon index and PD (phylogenetic diversity) index were calculated by *alpha_diversity.py.* Statistical tests were performed in R by employing standard functions and the “vegan” package (R Development Core Team, 2017). Data distribution and homogeneity of variance were determined by the Shapiro test, implemented in R (R Development Core Team, 2017). For determination of significant differences between treatments and samples, PERMANOVA analysis was performed with the “vegan” and “RVAideMemoire” packages in R. The “vegan” package was also used for calculation of distance matrices, clustering analysis and non-metric multidimensional scaling (NMDS) based on a weighted UniFrac distance matrix (Lozupone et al., 2011). For NMDS analysis, sample sequences were merged at core plot level with a resulting subsample size of 19,950.

For statistical analysis of abundance differences of single taxonomic groups between land use systems, normal distribution of values was tested first with Shapiro test in R. Depending on the result, normally distributed samples were analyzed with an ANOVA and Tukey test as *post hoc* tests. Non-normally distributed samples were tested by Kruskal–Wallis test and Pairwise Wilcoxon test as *post hoc* tests. Results were interpreted as significant with *p* < 0.05.

Identification of bacterial genera associated to the analyzed land use systems was performed by using the “Indicspecies” package in R. We calculated an abundance-based version of the phi coefficient of association, the point biserial correlation coefficient via the *multipatt* command based on abundance based OTU data (**Supplementary Table S2**). Prior to analysis, all OTUs belonging to the same genus were summarized. We visualized the associated taxa in a network that used the analyzed land uses as source nodes and the associated bacterial taxa as nodes, while the correlation coefficients were used as edges. Only taxa with significant correlation coefficients (*p* < 0.05) were included. Network generation was performed with Cytoscape version 3.5.1 by using the *edge-weighted spring embedded layout* algorithm, with edge width corresponding to the correlation coefficients and taxa abundance to node size.

Analysis of activity and metabolism was performed via functional predictions, which were calculated on version 123 of the SILVA database with the “Tax4Fun” package in R and visualized with the “gplots” package in R (Asshauer et al., 2015).

### Accession Numbers

The 16S rRNA transcript sequences were deposited in the National Center for Biotechnology Information (NCBI) Sequence Read Archive (SRA) under accession number PRJNA278020.

## Results and Discussion

### Impact of Different Land Use Systems on Active Soil Bacterial Community Composition

We analyzed a management gradient with increasing intensity from rainforest reference sites to jungle rubber over rubber plantations to oil palm plantations in two landscapes. The entire dataset comprised 1,333,275 high-quality 16S rRNA transcript sequences, which belonged to 32,280 different OTUs at species level (3% dissimilarity) (**Supplementary Tables S1, S2**). Species richness was highest in oil palm followed by rubber, rainforest, and jungle rubber (**Supplementary Figures S1, S2** and **Table S1**). Diversity indices Shannon and PD did not show significant differences between the analyzed land use systems (*p* > 0.11 and *p* > 0.06, respectively; **Supplementary Table S1**). Similar trends were observed in DNA-based studies targeting the conversion of rainforest to agricultural systems in which alpha and/or beta diversity increased as well, although the results were not completely consistent with respect to statistical significance (Carney et al., 2004; Tripathi et al., 2012; Schneider et al., 2015; Kerfahi et al., 2016).

**FIGURE 2:**
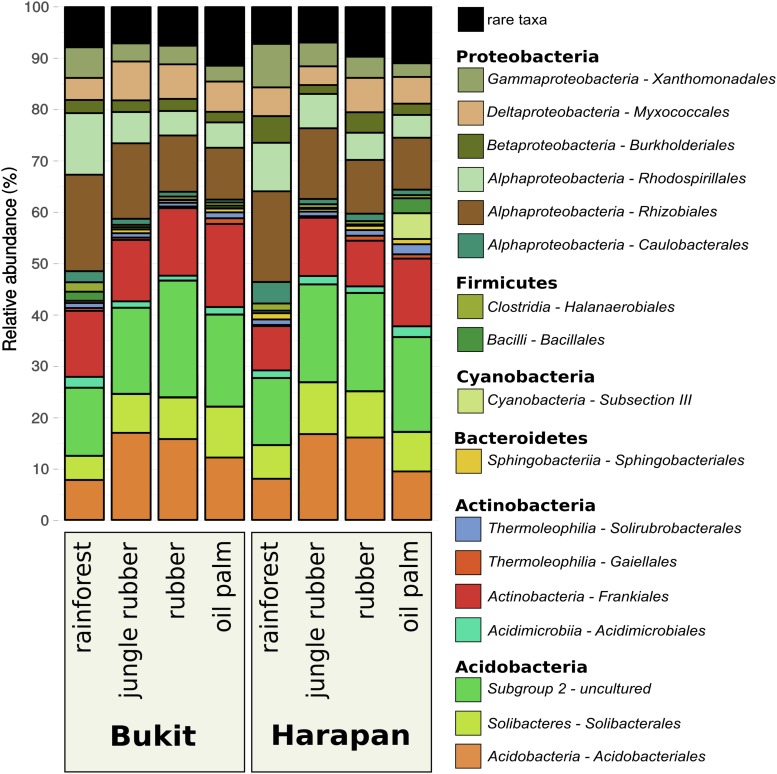
Community composition of active soil bacterial communities in three different land use systems and rainforest reference sites in two different landscapes. All plots were clustered according to their respective land use and landscape. Community compositions are displayed as relative abundances at order level based on 16S rRNA sequences obtained from extracted soil RNA. Taxa with abundances below 1% in each land use system were summarized as “rare taxa.” The detected orders are grouped to corresponding phylum (for details see **Supplementary Table S2**).

Composition of the active soil bacterial community showed similar patterns in the corresponding land use systems of both analyzed landscapes Bukit Duabelas and Harapan (**Figure 2**). *Proteobacteria* decreased continuously with increasing land use intensity in Bukit Duabelas and in Harapan (46.3 and 52.2% in rainforest to 29 and 28.3% in oil palm plantation, respectively), while the abundance of *Acidobacteria* showed a maximum in jungle rubber (42.8% in Bukit Duabelas and 47.6% in Harapan) and rubber systems (47.9% in Bukit and 46.7% in Harapan) before decreasing again in oil palm (42.5% in Bukit Duabelas and 36.9% in Harapan).

The different land use systems within the landscapes showed significant changes in the active bacterial community composition (*p* < 0.002; **Supplementary Table S3**). The abundances of *Rhizobiales* within the *Proteobacteria* decreased with increasing land use intensity (18.5% in rainforest to 10.45% in oil palm for Bukit Duabelas and 17.3% in rainforest to 10.6% in oil palm for Harapan), whereas *Frankiales* of the *Actinobacteria* increased (13% from rainforest to 15.6% in oil palm in Bukit Duabelas and from 8.6% in rainforest to 12.4% in oil palm in Harapan). Interestingly, *Acidobacteriales* and especially the *Acidobacteria* subgroup 2 showed an increase of abundance from rainforest (13.4% in Bukit Duabelas and 13% in Harapan) to rubber (22.6% in Bukit Duabelas and 19.6% in Harapan), which decreased in oil palm (17.9% in Bukit Duabelas and 18.1% in Harapan). *Proteobacteria* and *Acidobacteria* were reported to be very abundant at DNA level in the studied sites and in other locations (Rousk et al., 2010; Tripathi et al., 2012; Schneider et al., 2015). Here, at RNA level, *Proteobacteria* and *Acidobacteria* were the most abundant phyla as well. This is not a surprise, since *Acidobacteria* and *Alphaproteobacteria* were previously reported as generally high abundant in soils and important for decomposition of soil carbon (Hansel et al., 2008; Leff et al., 2012). *Proteobacteria* and *Acidobacteria* were reported to be very abundant at DNA level in the studied sites and in other locations (Rousk et al., 2010; Tripathi et al., 2012; Schneider et al., 2015). Here, at RNA level, *Proteobacteria* and *Acidobacteria* were the most abundant phyla as well. This is not a surprise, since *Acidobacteria* and *Alphaproteobacteria* were previously reported as generally high abundant in soils and important for decomposition of soil carbon (Hansel et al., 2008; Leff et al., 2012).

As mentioned before, the abundant *Rhizobiales*, which constitute the major part of detected *Proteobacteria*, decreased with increased land use intensity and increased fertilizer application from rainforest to oil palm plantation. As *Rhizobiales* are known to be involved in plant-associated and free-living N_2_ fixation, the higher availability of usable nitrogen compounds in fertilized systems reduce the requirement for microbial nitrogen fixation and favors other phylogenetic groups (Yoneyama et al., 2017). Furthermore, denitrification might be affected as well, since several taxa within the *Rhizobiales* were also reported to be involved in *nirK*-mediated denitrification (Bremer et al., 2007; Yoshida et al., 2009). In contrast, nitrification-related taxa like the *Nitrosomonadales* or *Nitrospira* increased from rainforest to rubber and oil palm plantations (relative abundances lower than 1%; data not shown). Additionally, it is notable that while *Proteobacteria* abundance decreased, *Acidobacteria* abundances (especially subgroup 2) increased, indicating negative correlations between these groups. Despite their high abundances in several studies, the ecological role of *Acidobacteria* in soil is still poorly understood. Some studies provide contradicting results in which positive as well as negative correlations as response to high nutrient input are mentioned for this taxon (Kielak et al., 2016). Interestingly, positive correlations between *Proteobacteria* and *Acidobacteria* have been shown as well, which is contrary to our results. This could be explained by the differences in acidobacterial subgroups detected in the other studies and the so far not completely understood roles of all subgroups within the *Acidobacteria* (Kielak et al., 2016).

### Effects of Abiotic Soil Parameters on Active Bacterial Communities

**FIGURE 3:**
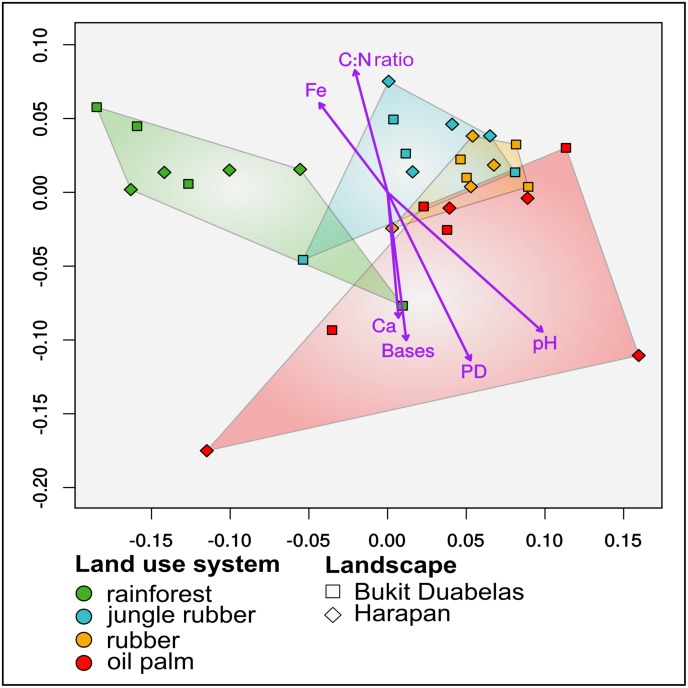
Non-metric multidimensional scaling (NMDS) based on a Weighted UniFrac distance matrix of soil bacterial community composition in all core plots of three converted land use systems (jungle rubber, rubber, and oil palm) and rainforest reference sites in two different landscapes. The detected stress level was 0.1415033 (for details see **Supplementary Figure S4**). The 96 subplot samples were merged to core plot level previous to calculation of the weighted UniFrac matrix. Purple arrows show significant correlations of abiotic measurements (carbon to nitrogen ratio (C:N), base saturation (bases), pH, calcium (Ca) and iron (Fe) and diversity metric PD (*p* < 0.05).

Shape and structure of prokaryotic communities are tightly connected with their surrounding environment and the corresponding abiotic and biotic environmental factors (Brevik et al., 2015). Environmental parameters are crucial factors for investigating soil-borne bacterial communities in agricultural systems (Rousk et al., 2010). Especially, the severe biodiversity and nutrient content alterations in agricultural land use systems compared to rainforest are of high importance for addressing and understanding the impact of rainforest conversion on microbial communities (Corre et al., 2006; Junier et al., 2010; Rousk et al., 2010; Dam et al., 2014). Based on non-metric multidimensional scaling (NMDS), differences in active community composition were tightly connected to conversion of rainforest to agricultural land use systems (**Figure 3**). Rainforest samples clustered separately from the converted systems. Additionally, clustering analysis confirmed that soil bacterial communities from rainforest core plots are distinct from that in almost all core plots of the converted systems (**Supplementary Figure S3**). Base saturation (*p* < 0.001, *r*^2^ 0.410) was one of the main drivers of active bacterial communities together with pH (*p* < 0.001, *r*^2^ 0.780), Fe content (*p* < 0.018, *r*^2^ 0.248), C:N ratio (*p* < 0.011, *r*^2^ 0.30) and Ca content (*p* < 0.037, *r*^2^ 0.2392). The two analyzed landscapes showed no significant difference in this respect. Base saturation, which displays soil fertility, exhibited a major impact on composition of active soil bacterial communities. Additionally, another study conducted on the same plots reported that base saturation was decreasing with increasing land use intensities, indicating that decreasing soil fertility has a major influence on active bacterial community structure (Allen et al., 2015). Soil pH is known as one of the major drivers for soil prokaryotic communities and pH changes were described as a common indirect consequence of fertilizer application in agricultural systems (Fierer et al., 2007; Foesel et al., 2014; Brenzinger et al., 2015; Herzog et al., 2015; Lammel et al., 2015; Stempfhuber et al., 2015; Zhalnina et al., 2015; Kaiser et al., 2016; Zhang et al., 2017). In our samples pH increased with higher land use intensity from rainforest to oil palm plantation. As biodiversity did not change significantly, pH might affect abundance of certain groups and consequently be the reason for the most prominent observed abundance changes within the *Alphaproteobacteria, Acidobacteriales*, and *Actinobacteria*.

Allen et al. (2015) suggested that a decreasing carbon to nitrogen ratio (C:N), which was identified as an additional significant driver for the active bacterial communities, typically indicates a shift toward a more bacteria-dominated system (Foesel et al., 2014; Allen et al., 2015). We identified C:N as a significant driver and observed for plantations compared to rainforest a decreasing C:N ratio in Harapan whereas the Bukit Duabelas samples showed similar values for all studied land use systems. Fe concentrations decreased from rainforest to the fertilized land use systems. Iron is often a limiting factor due to high demand not only of bacteria (Hibbing et al., 2009; Colombo et al., 2014). Thus, with decreasing iron content, we would expect a higher degree of bacterial competition and a community shaping effect of iron itself. Ca concentrations exhibited significant effects on active communities and showed a positive correlation with increasing land use intensity from rainforest to oil palm plantation. This is most likely connected to liming practices and therefore to fertilizer application to counteract soil acidification (Tripathi et al., 2012). Therefore, it is indicated that soil bacterial community shifts after rainforest conversion were caused by fertilizer application, liming and reduction of plant diversity as suggested in previous studies (Rooney and Kennedy, 2013; Abdi et al., 2016; Val-Moraes et al., 2016; Liu et al., 2017). Previously observed changes of soil parameters after rainforest transformation to oil palm and rubber plantations indicated that the availability of N and other nutrients rely on continuous fertilization and liming (Allen et al., 2015). Thus, it is likely that the observed active bacterial community structure is highly dependent on ongoing treatment such as fertilizer application and liming.

### Associations Between Specific Taxa of the Active Bacterial Community and Analyzed Land Uses

**FIGURE 4:**
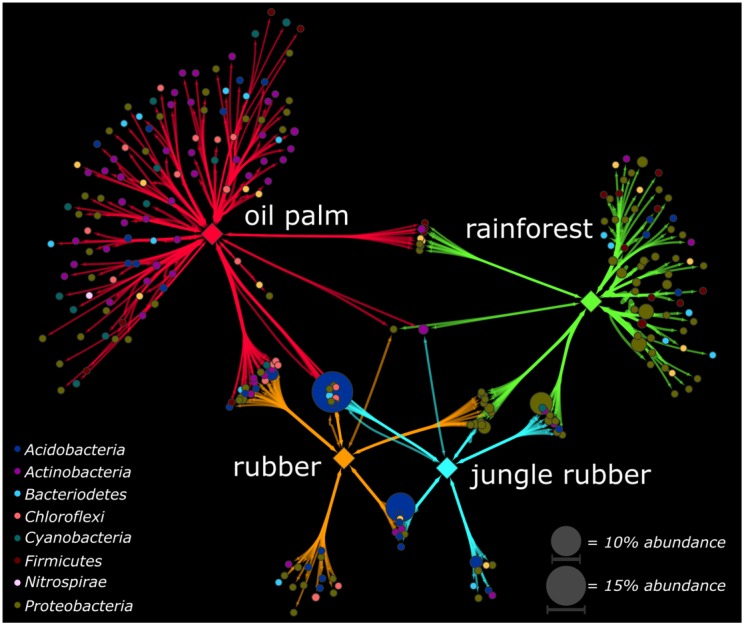
Association networks based on the point biserial correlation coefficient of genera related to the analyzed land use systems. Analyzed land use systems are displayed as hub nodes, while bacterial genera are displayed as nodes. Edges represent the point biserial correlation coefficient. Size of nodes and intensity of edges contribute to average abundance of genera in all land uses and weight of the respective point biserial correlation coefficient, respectively. Color of nodes contributes to prominent bacterial phyla. Hub nodes and edges are colored according to analyzed land use systems rainforest, jungle rubber, rubber, and oil palm. Only significant associations were included in the analysis (*p* < 0.05).

To identify genera, which were significantly associated with the analyzed land use systems and are suitable as indicators for one or combinations of land use systems, we calculated the point biserial correlation coefficient for each genus, which is the abundance-based counterpart of the phi coefficient. We detected for 270 (24%) of the 1,124 in total detected genera significant biserial correlation coefficients (*p* < 0.05). Most of these genera were associated to oil palm plantation (153 genera associated in total, 113 genera exclusively to oil palm) and rainforest (95 genera in total, 62 genera exclusively to rainforest). Furthermore, the point biserial correlation values and correspondingly the strength of association to the respective system were highest in oil palm plantation and rainforest compared to jungle rubber and rubber plantation (**Supplementary Figure S5**). The majority of associated genera in oil palm and rainforest belonged to the *Actinobacteria* (oil palm) and *Proteobacteria* (rainforest) (**Figure 4**). This is in accordance to our analysis of community composition in which *Proteobacteria* decreased from rainforest to oil palm plantation whereas *Actinobacteria* increased.

Strongest unique associations of rainforest were detected for genera within the *Rhizobiales* (*Beijerinckiaceae*), *Rhodospirillales* (*Acetobacteraceae*), and *Caulobacterales* (*Caulobacteraceae*). Since members of the *Rhizobiales* or *Rhodospirillales* like *Beijerinckiaceae* are associated with nitrogen fixation, the strong association to rainforest indicates a higher importance of nitrogen fixation for gaining usable nitrogen than in fertilized land use systems. Associations were also detected for *Rickettsiales*, which are also known for endosymbiotic relationships with eukaryotes (Taylor et al., 2012). This could be an indication for the higher biodiversity outside the bacterial domain in rainforests. The association of the acidobacterial subgroup 2 to rainforest is surprising, since its abundance increased from rainforest toward the studied agricultural land use systems. Except for one genus, all associations of genera within the *Clostridia* were exclusively to rainforest. Their ability to fix nitrogen in anaerobic environments might explain this when assuming a higher demand of nitrogen fixation in rainforest soils compared with agricultural managed soils (Hayat et al., 2010). Nitrogen-fixing clostridia were also reported as dominant in Amazonian rainforest soils, but contrary results have been reported for other tropical forest soils like the Brazilian Atlantic forest. Thus, these results indicate a specific association to soil properties rather than a general affiliation to tropical forest soils (Faoro et al., 2010).

In oil palm, the majority of associated species belonged to the *Actinobacteria*, followed by *Proteobacteria*. *Actinobacteria* are reported as tolerant to higher temperatures, as well as *Chloroflexi*, which were exclusively detected as associated taxa for oil palm (Bouskill et al., 2012). It has been proposed that higher light availability in oil palm plantations compared to rainforest, which also results in higher soil temperatures, affect the soil microbiome (Schneider et al., 2015). We detected the nitrification-related genus *Nitrospirales* and two genera of the *Nitrosomonadales* exclusively in oil palm plantations as associated taxa (Lücker et al., 2010; Ma et al., 2013). This indicates higher degrees of nitrification in oil palm soils compared to rainforest, probably caused by fertilizer application and correspondingly nitrogen input, leading to more favorable conditions for nitrifiers (Ma et al., 2013; Quan et al., 2016).

Interestingly, the detected genera with highest abundance belonged to *Acidobacteria* and were associated with combinations of land use systems. An uncultured genus within the subgroup 2 with an average abundance of 14.9% in all land use systems was associated with a combination of jungle rubber, rubber, and oil palm while an uncultured genus from subgroup 1 with an average abundance of 9.8% was characteristic for the combination of jungle rubber and rubber. In general, *Acidobacteriaceae* increased in abundance from rainforest to oil palm, with maxima in jungle rubber and rubber, indicating specific adaptations to these environments. The ecological role of the numerous subgroups within the *Acidobacteria* is under discussion. It was reported that the abundance of some subgroups increased during Amazonian rainforest conversion to managed soils whereas that of other subgroups decreased (Navarrete et al., 2015).

Overall, we could show that patterns of associated genera for different land use types and corresponding soil properties are distinguishable. The observed pattern corresponded to our other results, indicating a shift from higher abundances and associations of nitrogen fixation-related taxa in rainforest to conditions more favorable for groups associated with nitrification and heat-tolerance in plantations. This indicated a general shift of bacterial functions within these systems from higher importance of biological nitrogen acquisition in unfertilized systems to higher degrees of nitrification in fertilized land use systems.

### Relationship Between Rainforest Conversion and Key Functions of the Bacterial Community

**FIGURE 5:**
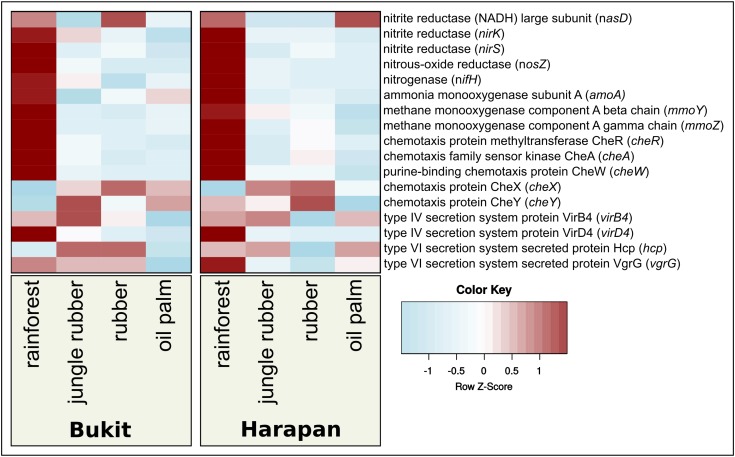
Prediction-based abundance of selected key genes in rainforest and converted land use systems. The selected genes were used as markers for genes involved in nitrogen cycling, methane oxidation, chemotaxis, and type IV and type VI secretion systems.

Besides shape and structures of soil bacterial communities, functional profiles and measures of activity are necessary to study the bacterial responses. To obtain these profiles and activity measurements in ecological studies are often a challenge, since metatranscriptomes and high sample numbers are needed, which can be challenging in large scale projects and areas with infrastructure gaps. An alternative, especially for large sample numbers, are functional predictions based on 16S rRNA analysis using bioinformatic tools like “Tax4Fun” (Asshauer et al., 2015; Koo et al., 2017) or “PICRUSt” (Langille et al., 2013). It was shown that these tools provide a sufficient accuracy of functional profiles compared to those derived directly from metagenomic or metatranscriptomic sequence analyses. These tools are frequently used in various projects (Langille et al., 2013; Asshauer et al., 2015; Mukherjee et al., 2017; Wemheuer et al., 2017). In this study, we used Tax4Fun to investigate bacterial metabolic activity (**Figure 5**) and focused on all pathways that showed an abundance of at least 1 % (**Supplementary Table S4**). Within the thereby recovered 27 KEGG categories, relative abundances ranged from 1 to 8% (data not shown). We observed different patterns of predicted gene abundances within the analyzed land use systems in both landscapes, which were expected due to their different properties. Since previous results showed relations of bacterial community composition and fertilizer application, as well as bacterial community associations and land use changes, we analyzed the changes of relative abundances of predicted genes encoding marker enzymes for selected metabolic traits, e.g., nitrogen metabolism (**Supplementary Table S5**). We analyzed predicted gene abundances of the ammonium monooxygenase subunit A (*amoA*) for nitrification, nitrogenase (*nifH*) for nitrogen fixation, nitrous oxide reductase (*nosZ*) for denitrification and assimilatory nitrite reductase (*nasD, nirK* and *nirS*) for nitrate/nitrite assimilation (Hai et al., 2009; Pappu et al., 2017). For methane-related metabolism, we analyzed the gene encoding the methane monooxygenase (*mmoY* and *mmoZ*) (Henckel et al., 1999; Murrell et al., 2000; Sengupta and Dick, 2017). In order to address bacterial interactions, we analyzed the chemotaxis related genes *cheA, cheW, cheY, cheX*, and *cheR* and for secretion systems the type IV secretion system genes *virB4* and *virD4* and the type VI secretion system genes *hcp* and *vgrG* (Cascales and Cambillau, 2012; Guglielmini et al., 2014; Briegel et al., 2015; Jones and Armitage, 2015; Zinicola et al., 2015; Fedi et al., 2016).

In both landscapes, predicted abundances for nitrogenase and nitrous oxide reductase gene abundances were significantly different between rainforest and converted systems (**Figure 5**). We observed highest numbers in rainforest and lowest in converted systems (*p* < 0.05). Predicted gene abundances for *nasD, nirK* did not show significant differences (*p* > 0.05). The predicted gene abundances for *mmoA* exhibited significant higher abundances in rainforest compared to jungle rubber and plantations (*p* < 0.0002).

The gene of the nitrous oxide reductase (*nosZ*) abundances was significantly higher in rainforest samples compared to transformed systems (*p* < 0.0001). Deduced abundances for the nitrite reductase gene *nirS* showed significant decreases from rainforest to all other studied land use systems in Harapan landscape (*p* < 0.02), but no significant changes in Bukit Duabelas Landscape. In contrast to Bukit Duabelas landscape, *amoA* showed significantly higher abundance in Harapan rainforest, while analyzed methane monooxygenases did not show significant differences (**Supplementary Table S5**).

Higher abundance of nitrogenase in rainforest suggests, that biological nitrogen uptake through nitrogen fixation is decreased in converted systems, which are in accordance to the recorded suppressed biological nitrogen fixation in converted systems (Corre et al., 2006; Pajares and Bohannan, 2016). We assume that the additional input of ready to use nutrients in agricultural systems is less beneficial for soil bacterial groups with the ability to fix nitrogen, resulting in lower abundances of nitrogen fixation related taxa like *Rhizobiales* in fertilized systems (Corre et al., 2006; Barron et al., 2009; Isobe et al., 2012; Waring et al., 2014; Cong et al., 2015; Pajares and Bohannan, 2016). Interestingly, *Alphaproteobacteria*, which include the *Rhizobiales*, were negatively affected as well by rainforest conversion as shown in **Figure 2**. In Harapan landscape, predicted abundances for genes involved nitrification were lower in converted systems as well. Methane metabolism is known to be linked to nitrogen metabolism, due to similarities between the ammonia monooxygenase and the methane monooxygenase (Henckel et al., 1999; Murrell et al., 2000; Sengupta and Dick, 2017). Both enzymes catalyze similar reactions and the corresponding bacterial groups bear the potential to outcompete each other (Akiyama et al., 2014; Zheng et al., 2014). Accordingly, the methane monooxygenase gene abundance was higher in rainforest than that of ammonia monooxygenase subunit A gene. Additionally, the predicted gene abundances for denitrification were higher in rainforest compared to the other studied systems in both landscapes. Therefore, we assume that in converted systems with higher nitrogen disposition and availability, active soil bacterial communities respond to land use management and higher nutrient input with decreased nitrogen fixation. A decrease in nitrification and denitrification seems unlikely though, since previous studies demonstrated the increase of the activity of these processes under the influence of fertilizer input (Ma et al., 2013; Quan et al., 2016; Wang et al., 2018). Additionally, we observed that taxa which are associated with the above-mentioned nutrient cycling pathways underwent the most drastic changes in relative abundance as well. Indicator species analysis showed that genera with nitrification ability such as *Nitrospira* and *Nitrosomonadales* were detected in oil palm soils (**Figure 4**). However, since functional prediction cannot assign all taxa with their respective functional potential due to a lack of detailed information about certain groups (e.g., subgroups of *Acidobacteria*), additional analysis is required in this specific case (Kielak et al., 2016).

Abundances of chemotaxis genes *cheW* and *cheR* were highest in rainforest and decreased toward higher land use intensity (all *p*-values < 0.05). However, not all observed differences of the selected chemotaxis-related genes were highest in rainforest. The *cheX* gene abundance was lowest in rainforest and highest in plantations (*p* < 0.05). No significant differences were observed for sensor kinase *cheA* and response regulator *cheY*. For all tested chemotaxis-related genes, except *cheA* and *cheY*, we detected significant differences between rainforest and plantations. The analyzed *che* genes are part of the same operon and were shown to be connected to swarming capacity and especially in pathogens (Lambert et al., 2015; Fedi et al., 2016). Since we observed significant differences between rainforest and the other studied land use systems for almost all tested genes, we assume that rainforest transformation not only affects nutrient recycling but also interactions. Additionally, it is possible that due to strong association of the analyzed *che* genes with pathogenic lifestyles, community dynamics might be altered regarding pathogenicity as well.

The abundance changes of the investigated secretion system-related functions indicated an impact of rainforest conversion on interspecific bacterial activity. Type IV secretion system genes showed significant higher abundance in rainforest compared to oil palm samples (*p* < 0.05). We conclude that exchange of nucleic acids between bacteria is decreased in nutrient-rich managed land use systems, possibly due to a lower degree of competition resulting in a lower pressure for adaptations and hence nucleic acid exchange. Type VI secretion system genes showed slightly higher abundance in rainforest compared to oil palm (*p* < 0.05) as well. These results hint less negative interaction and competition by pathogenesis via antibacterial compounds (Tripathi et al., 2016). In contrast, we previously observed a significant effect of iron content on the active bacterial community composition, which is likely caused by competition for this limiting compound. However, we did not observe similar trends for all analyzed predicted genes regarding interaction and competition, indicating the need for further analysis. We recorded several significant changes for the studied functions, but it has to be considered that prediction-based methods only provide indications, which have to be confirmed by additional analysis such as full metagenome and metatranscriptome analyses.

## Conclusion

We confirmed our first hypothesis (a) that the diversity of the active bacterial community was not significantly affected by rainforest conversion. We showed that rainforest transformation has a significant impact on active bacterial community composition as suggested in hypothesis (b). Furthermore, we could show that changes in soil characteristics deriving from rainforest conversion and management are a major factor in reshaping the active bacterial community. Additionally, we identified that change in pH, base saturation, Fe content and C:N ratio is significant drivers of soil bacterial community composition. This suggests a direct connection to fertilizer applications and liming, which affects composition and amount of available nutrients, i.e., nitrogen-containing compounds. Rainforest soils and converted systems revealed distinguishable patterns of associated taxa, which illustrate the changed requirements for bacterial life in the different land use systems as mentioned in hypothesis (c). Furthermore, predicted functional profiles revealed that uptake of nutrients like nitrogen through biological fixation decreases with higher land use intensity. It was also indicated that interactions in form of nucleic acid exchange as well as antagonistic or competitive behavior were reduced after rainforest conversion and it is likely that rainforest transformation leads to soil bacterial communities with severely altered nutrient cycling activity. Thus, active bacterial communities are significantly affected by rainforest transformation. In addition to the impact on active community composition, we could show that changes of soil properties introduced by management (e.g., fertilizer application) are the main drivers for adaptations and probably changes in bacterial functioning.

## Author Contributions

RD designed and conceived the study. Soil sampling for prokaryotic community analysis was performed by ME, MW, and AM. DS, MH, and ME carried out the field and laboratory work. DB and DS prepared and analyzed the data. All authors interpreted the results and wrote the paper.

## Conflict of Interest Statement

The authors declare that the research was conducted in the absence of any commercial or financial relationships that could be construed as a potential conflict of interest.
