# Supplementary material for: How Rainforest Conversion to Agricultural Systems in Sumatra (Indonesia) Affects Active Soil Bacterial Communities
Source: Front Microbiol. 2018 Oct 10;9:2381. doi: 10.3389/fmicb.2018.02381 (PMC6191527; doi:10.3389/fmicb.2018.02381)
Supplement: Supplementary file 6 [file Data_Sheet_1.PDF]

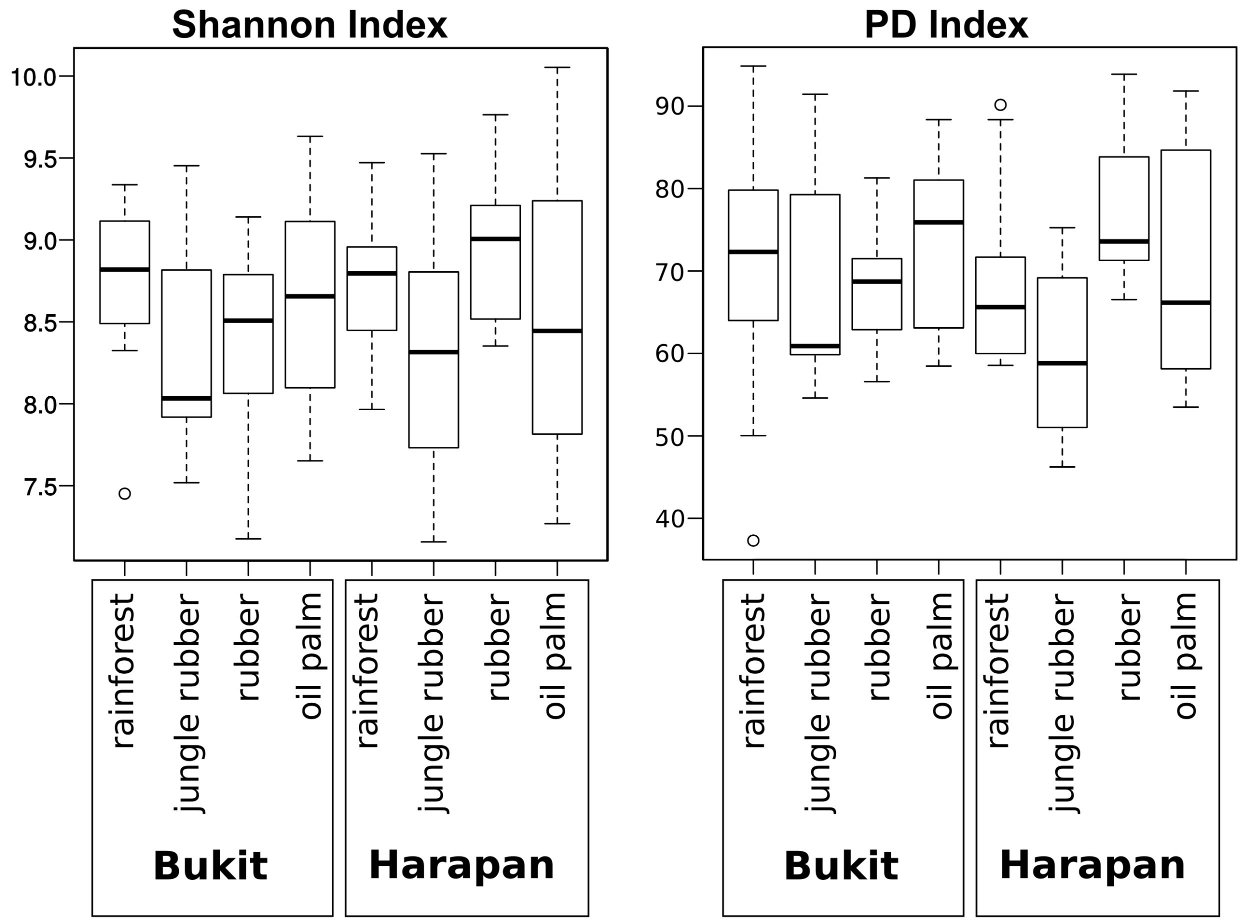

Figure S1. Average Shannon and PD diversity indices. Values were summarized from all subplot values at land use level in the corresponding landscape.
