# Supplementary figures and images for: How Rainforest Conversion to Agricultural Systems in Sumatra (Indonesia) Affects Active Soil Bacterial Communities
Source: Front Microbiol. 2018 Oct 10;9:2381. doi: 10.3389/fmicb.2018.02381 (PMC6191527; doi:10.3389/fmicb.2018.02381)

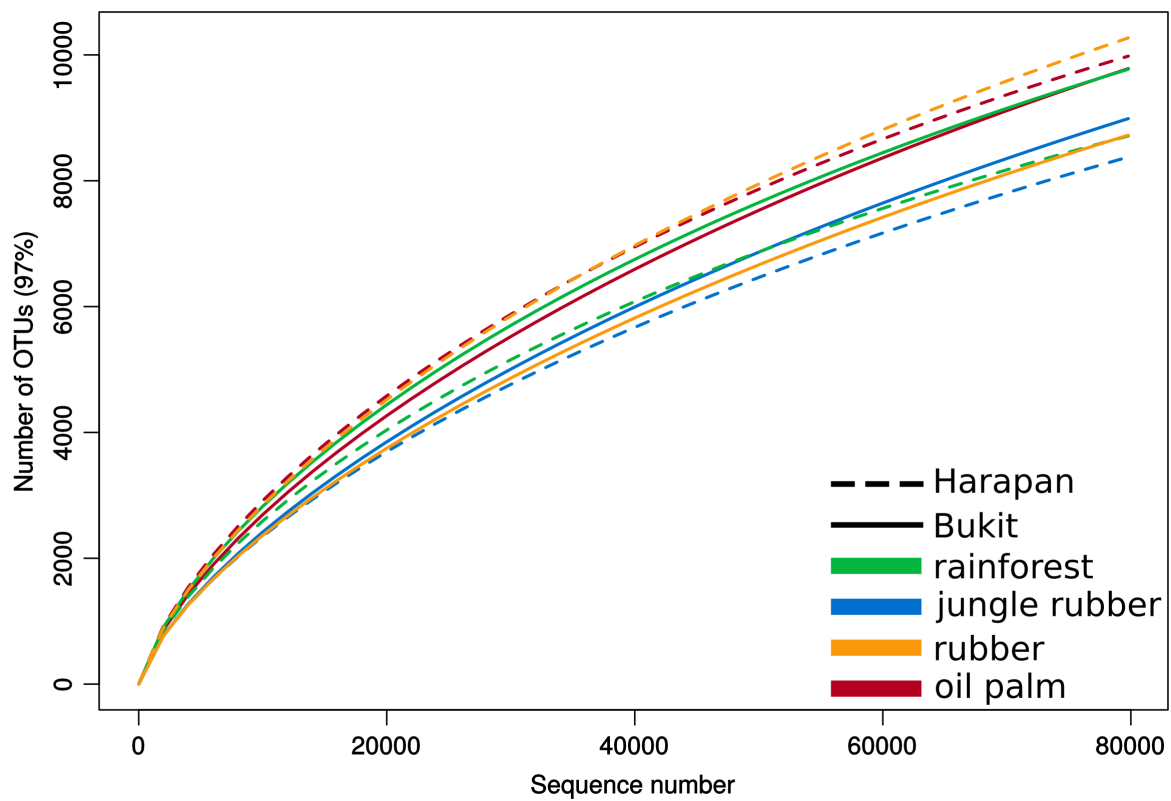

**Figure S2.** Rarefaction curves for landscape level and land use systems.

Supplement: Supplementary file 7 [file Data_Sheet_2.PDF]

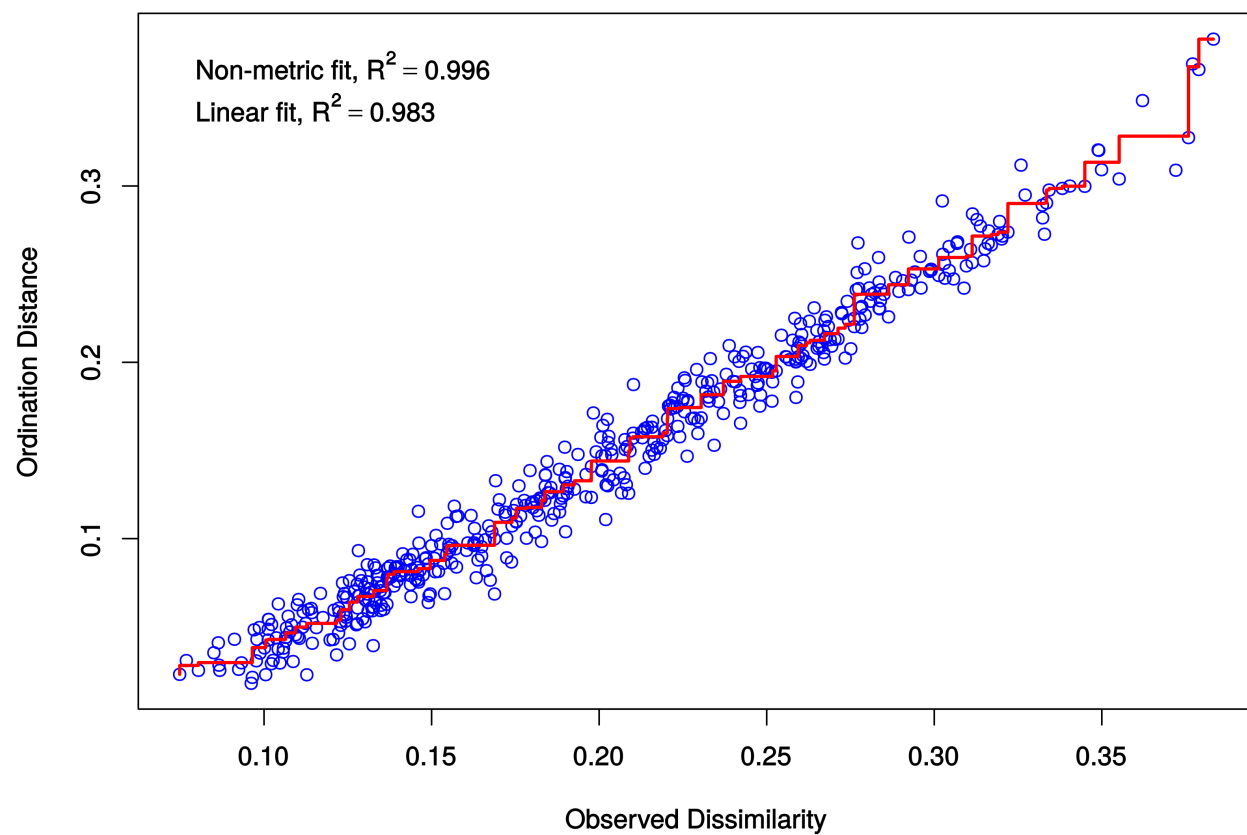

**Figure S4.** Stressplot for calculated NMDS shown in Figure 3 based on weighted UniFrac.

Supplement: Supplementary file 9 [file Data_Sheet_4.PDF]
