# Supplementary material for: How Rainforest Conversion to Agricultural Systems in Sumatra (Indonesia) Affects Active Soil Bacterial Communities
Source: Front Microbiol. 2018 Oct 10;9:2381. doi: 10.3389/fmicb.2018.02381 (PMC6191527; doi:10.3389/fmicb.2018.02381)
Supplement: Supplementary file 8 [file Data_Sheet_3.PDF]

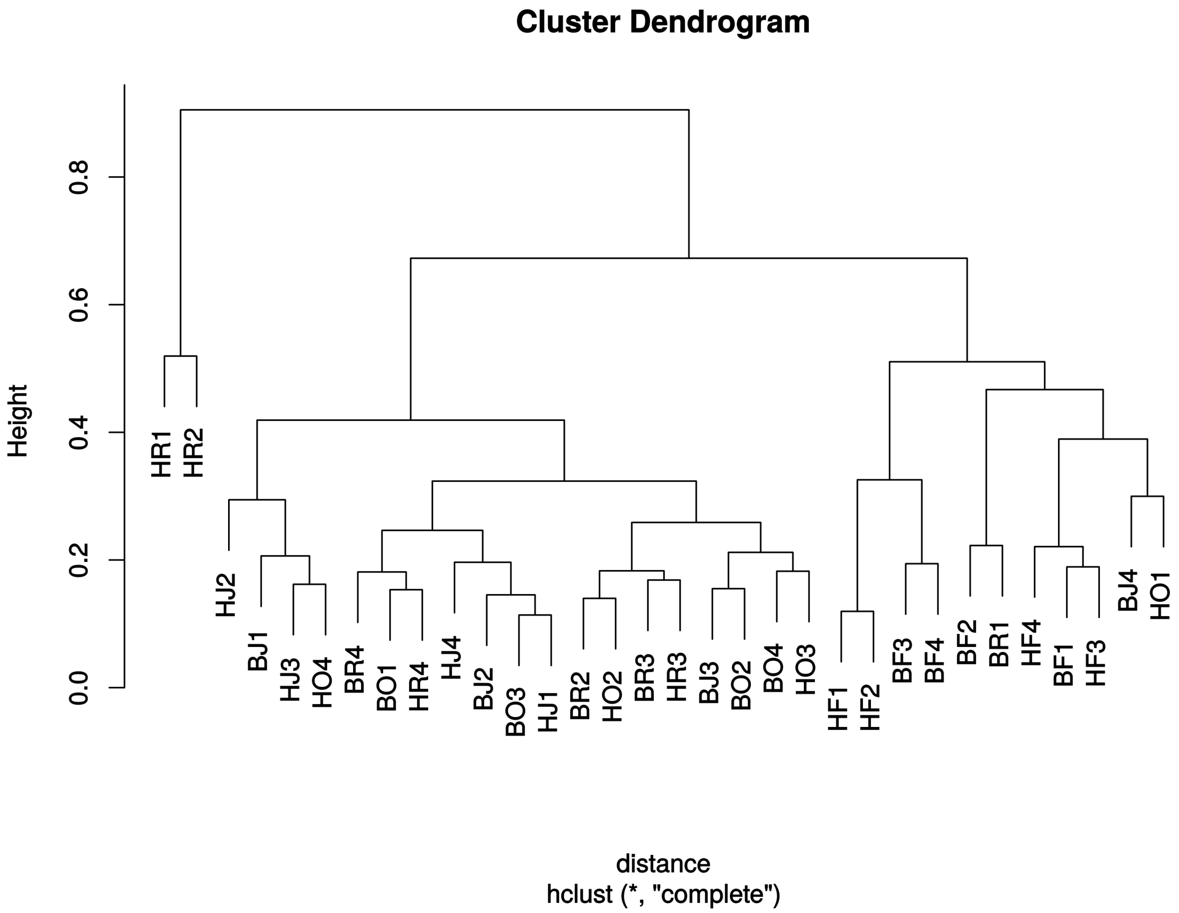

Figure S3. Clustering analysis of used samples on core plot level. Cluster analysis was performed by using hclust.
