# Supplementary material for: How Rainforest Conversion to Agricultural Systems in Sumatra (Indonesia) Affects Active Soil Bacterial Communities
Source: Front Microbiol. 2018 Oct 10;9:2381. doi: 10.3389/fmicb.2018.02381 (PMC6191527; doi:10.3389/fmicb.2018.02381)
Supplement: Supplementary file 10 [file Data_Sheet_5.PDF]

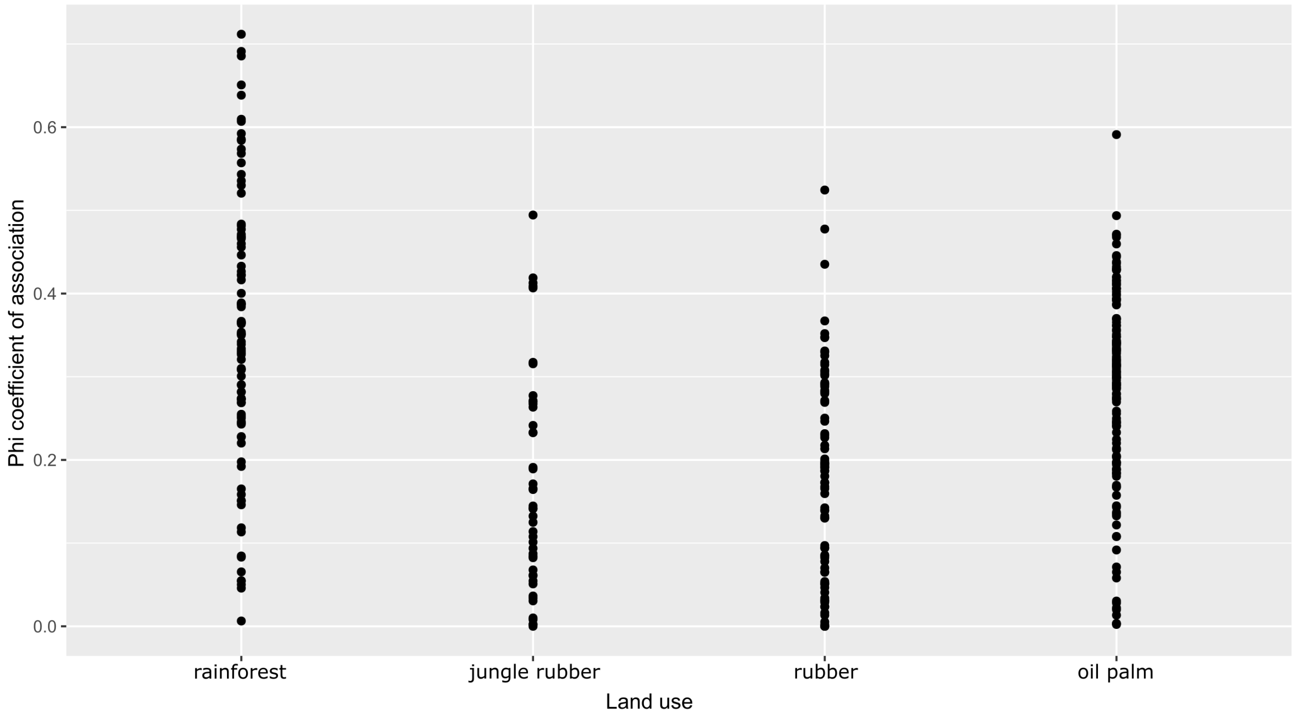

Figure S5. Distribution of obtained point biserial correlation coefficients. Each dot displays one obtained value in the respective land use systems. Only significant values were included ($p < 0.05$).
